# Crystal structure of tetra­kis­(isonicotinamide-κ*N*)bis­(thio­cyanato-κ*N*)cobalt(II)–isonicotinamide–ethanol (1/2/1)

**DOI:** 10.1107/S2056989016010951

**Published:** 2016-07-12

**Authors:** Tristan Neumann, Inke Jess, Christian Näther

**Affiliations:** a Institut für Anorganische Chemie, Christian-Albrechts-Universität Kiel, Max-Eyth Strasse 2, D-24118 Kiel, Germany

**Keywords:** crystal structure, hydrogen bonding, isonicotinamide, cobalt

## Abstract

The asymmetric unit of the title compound, [Co(NCS)_2_(C_6_H_6_N_2_O)_4_]·2C_6_H_6_N_2_O·C_2_H_5_OH, comprises one Co^II^ cation, two thio­cyanate anions, four coordinating and two solvent isonicotinamide molecules and one ethanol solvent mol­ecule. The Co^II^ cations are octa­hedrally coordinated by four N-coordinating isonicotinamide ligands and two terminally N-bonded thio­cyanate anions. These discrete complexes are linked by inter­molecular N—H⋯O and N—H⋯S hydrogen-bonding inter­actions into a three-dimensional network. The two isonicotinamide and the ethanol solvent mol­ecules are embedded in channels of this network and are linked through further N—H⋯O and N—H⋯N hydrogen bonds to the network. The ethanol solvent mol­ecule is disordered over two sets of sites (occupancy ratio 0.6:0.4).

## Abstract 2

The crystal structure of the title compound consists of discrete octa­hedral cobalt(II) complexes that are linked by a variety of hydrogen-bonding inter­actions into a three-dimensional network.

## Chemical context   

There is an increasing inter­est in compounds showing cooperative magnetic properties, such as ferromagnetism, anti­ferromagnetism and metamagnetism or a slow relaxation of the magnetization, indicative of single-mol­ecule or single-chain magnetism (Gao *et al.*, 2009 ▸; Ma *et al.*, 2009 ▸; Palion-Gazda *et al.*, 2015 ▸; Näther *et al.*, 2013 ▸). In this context we have reported on a number of one-dimensional cobalt(II) thio­cyanate coordination compounds with different N-donor co-ligands that show slow relaxations of the magnetization which in some compounds can be traced back to the behaviour of single-chain magnets (SCM) (Wöhlert *et al.*, 2014 ▸; Werner *et al.*, 2014 ▸, 2015*a* ▸,*b* ▸,*c* ▸,). In the course of our systematic investigation of these materials, we became inter­ested in the monodentate ligand isonicotinamide, which can coordinate with the N atom to the Co^II^ atoms, forming the desired one-dimensional compounds. However, instead of the expected chain compound, a discrete complex with additional solvate mol­ecules of composition [Co(NCS)_2_(C_6_H_6_N_2_O)_4_]·2C_6_H_6_N_2_O·C_2_H_5_OH was obtained in the current study and characterized by single-crystal X-ray diffraction.

## Structural commentary   

**Figure 1:**
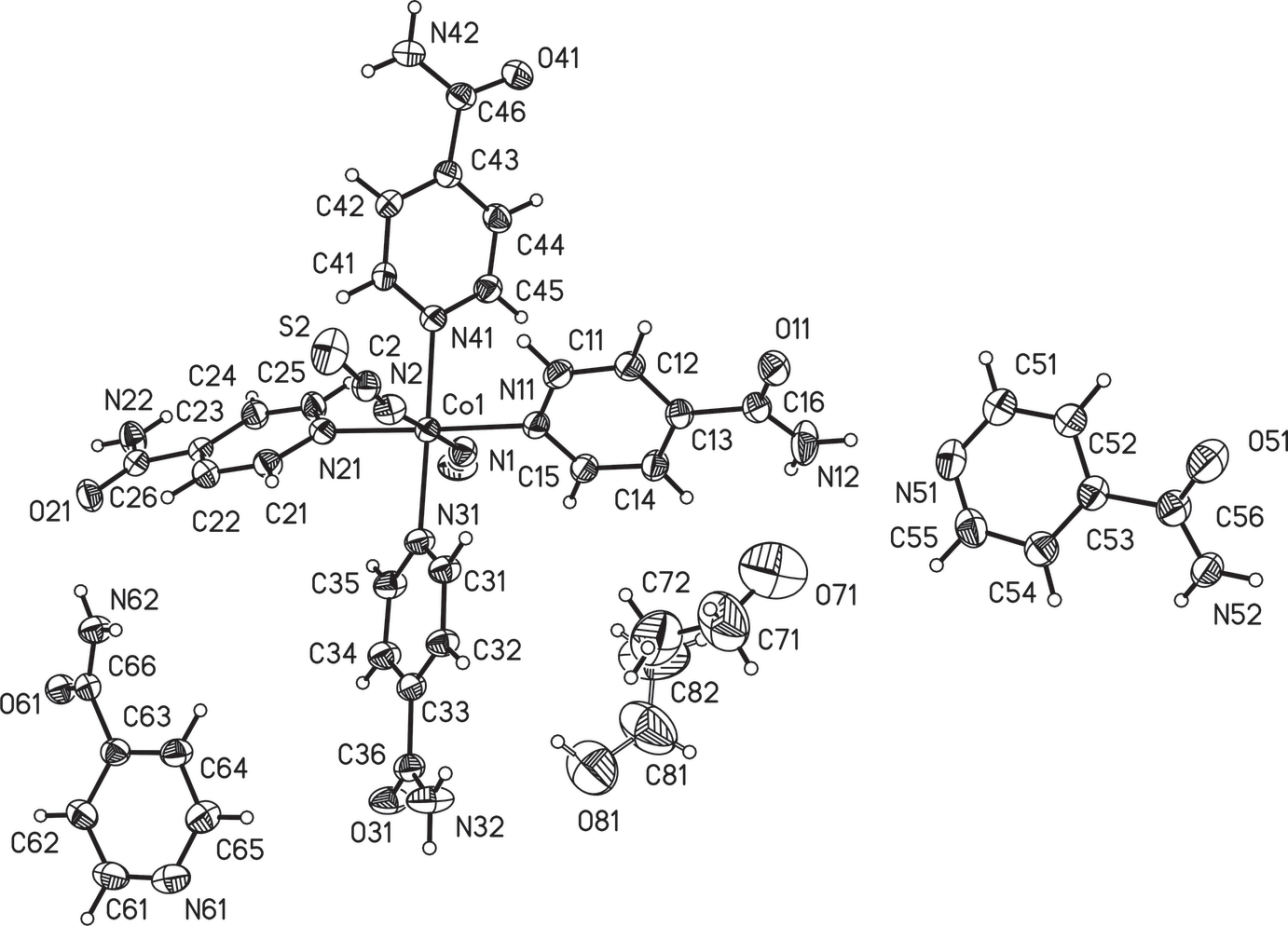
View of the asymmetric unit of the title compound, with atom labelling and displacement ellipsoids drawn at the 50% probability level. The positional disorder of the ethanol mol­ecule is shown by full and open bonds for the two orientations.

The asymmetric unit of the title compound consists of one Co^II^ cation, two thio­cyanate ligands, six isonicotinamide mol­ecules (four coordinating, two non-coordinating) and one positionally disordered ethanol solvent mol­ecule. The Co^II^ cation is coordinated by two terminal N-bonded thio­cyanate anions and four N-coordinating isonicotinamide ligands, forming a slightly distorted octa­hedron (Fig. 1 ▸). Bond lengths [Co—N range: 2.074 (3)–2.185 (2) Å] and angles [N—Co—N range: 88.09 (9)–91.91 (10)° for *cis* and 177.27 (10)–178.32 (11)° for *trans* angles] are indicative for a slight distortion and are comparable with those in similar coordination compounds with Co^II^, thio­cyanate anions and N-bound co-ligands.
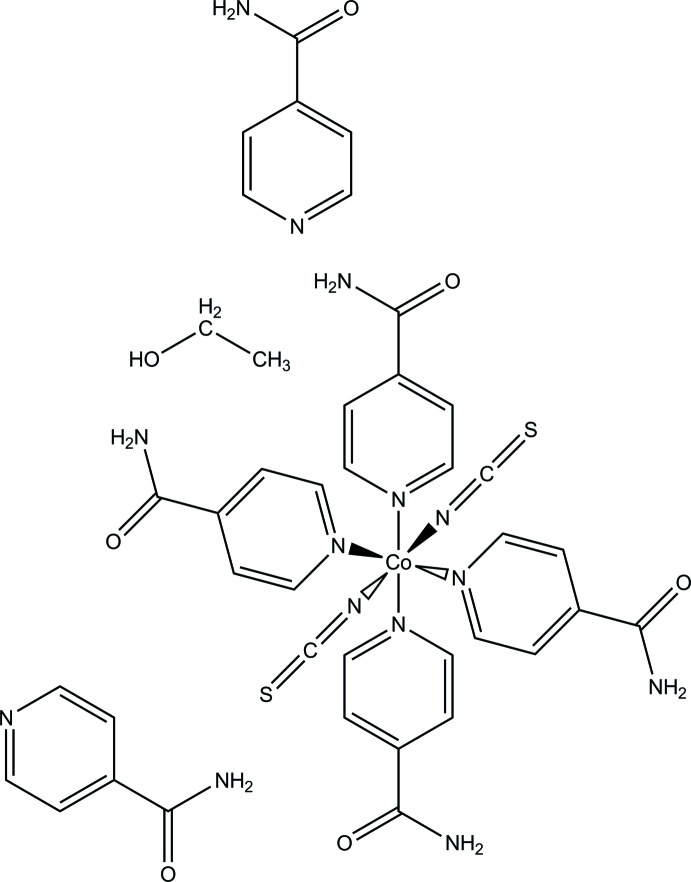


## Supra­molecular features   

**Figure 2:**
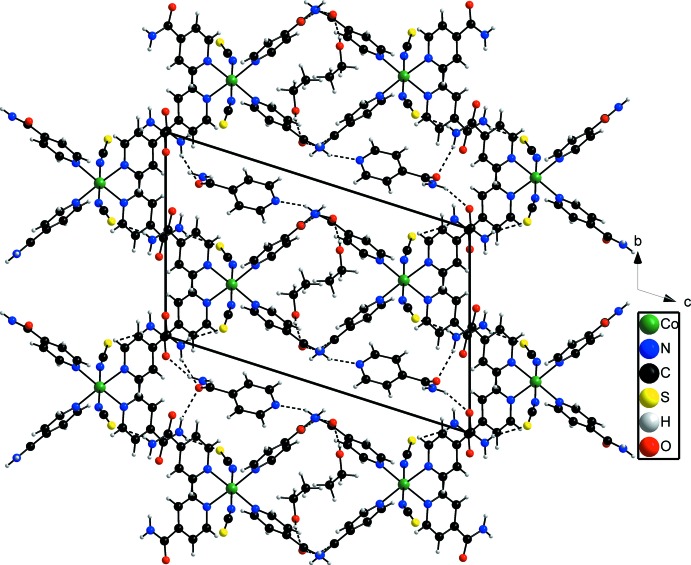
Crystal structure of the title compound in a view along the *a* axis. Inter­molecular hydrogen bonding is shown as dashed lines and the second orientation of the disordered ethanol mol­ecule is omitted for clarity.

**Figure 3:**
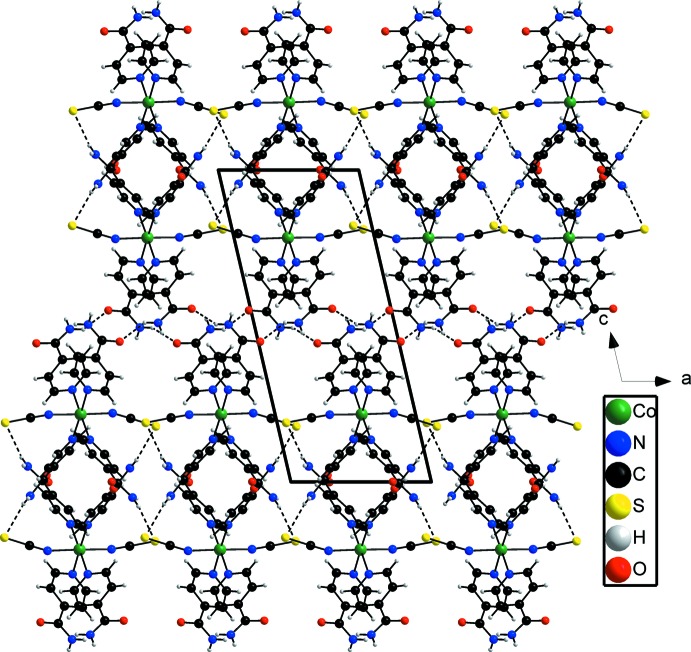
Crystal structure of the title compound in a view along the *b* axis. Inter­molecular hydrogen bonding is shown as dashed lines and the second orientation of the disordered ethanol mol­ecule is omitted for clarity.

In the crystal structure of the title compound, neighboring complexes are linked into chains extending along the *a* axis by inter­molecular N—H⋯O hydrogen-bonding inter­actions (Fig. 2 ▸, Table 1 ▸). These chains are further linked into a three-dimensional network by inter­chain N—H⋯S hydrogen bonding between the thio­cyanate anions and the amide H atoms of neighboring complexes (Fig. 3 ▸, Table 1 ▸). In this way, two types of channels are formed along the *a* axis. In the larger channels, the isonicotinamide solvent mol­ecules are embedded whereas the smaller channels are occupied by the disordered ethanol mol­ecules (Figs. 2 ▸ and 3 ▸). The solvent mol­ecules are linked by O—H⋯O, N—H⋯O and N—H⋯N hydrogen-bonding inter­actions to the the isonicotinamide ligands that form the channels. Weak C—H⋯O and C—H⋯S inter­actions are also observed, consolidating the packing of the crystal structure.

## Database survey   

In the Cambridge Structure Database (Version 5.37, last update 2015; Groom *et al.*, 2016 ▸) only five structures of coordination compounds with isonicotinamide and thio­cyanate as ligands are reported: two clathrates of nickel coordination polymers, in which the metal atoms are connected into chains by μ-1,3-bridging thio­cyanate ligands of which one contains 9,10-anthra­quinone and the other pyrene as clathrate mol­ecules (Sekiya *et al.*, 2009 ▸). Furthermore, a one-dimensional cadmium 9,10-di­chloro­anthracene-clathrate with bridging μ-1,3-thio­cyanate ligands between the metal atoms is reported (Sekiya & Nishikiori, 2005 ▸), as well as a three-dimensional network consisting of cadmium cations with μ-1,3-bridging thio­cyanate ligands (Yang *et al.*, 2001 ▸), and finally one Cu coordination polymer in which Cu–NCS sheets are observed (Đaković *et al.*, 2010 ▸). In this context we have reported recently on a Zn complex in which the Zn cations are tetra­hedrally coordinated by two terminal N-bonded thio­cyanate anions and two isonicotinamide ligands (Neumann *et al.*, 2016 ▸).

## Synthesis and crystallization   

Cobalt(II) thio­cyanate and isonicotinamide were obtained from Alfa Aesar and were used without further purification. Single crystals suitable for structure analysis were obtained by the reaction of 26.3 mg Co(NCS)_2_ (0.15 mmol) with 73.3 mg isonicotinamide (0.6 mmol) in ethanol (1.5 ml) after being allowed to stand for a few days at room temperature.

## Refinement   

Crystal data, data collection and structure refinement details are summarized in Table 2 ▸. The C—H, O—H and N—H hydrogen atoms were located in a difference map but were positioned with idealized geometry (methyl and O—H hydrogen atoms were allowed to rotate but not to tip) and were refined with *U*_iso_(H) = 1.2*U*_eq_(C,N) (1.5 for methyl and O—H hydrogen atoms) using a riding model with C—H = 0.95 Å for aromatic, C—H = 0.98 Å for methyl, N—H = 0.88 Å and O— H = 0.84 Å, respectively. The ethanol mol­ecule was found to be disordered over two sets of sites and was refined with fixed occupation factors of 0.6 and 0.4, respectively.

## Supplementary Material

Crystal structure: contains datablock(s) I. DOI: 10.1107/S2056989016010951/wm5305sup1.cif

Structure factors: contains datablock(s) I. DOI: 10.1107/S2056989016010951/wm5305Isup2.hkl

CCDC reference: 1491089

Additional supporting information: crystallographic information; 3D view; checkCIF report

## Figures and Tables

**Table 1:** Hydrogen-bond geometry (Å, °)

*D*—H⋯*A*	*D*—H	H⋯*A*	*D*⋯*A*	*D*—H⋯*A*
C11—H11⋯S1^i^	0.95	3.03	3.676 (3)	127
C14—H14⋯O31^ii^	0.95	2.62	3.532 (4)	162
C15—H15⋯O81^ii^	0.95	2.60	3.454 (7)	149
N12—H12*A*⋯N51	0.88	2.09	2.936 (4)	160
N12—H12*B*⋯O31^ii^	0.88	2.12	2.879 (4)	144
C25—H25⋯O41^iii^	0.95	2.47	3.100 (4)	124
N22—H22*A*⋯S2^iv^	0.88	2.60	3.439 (3)	160
N22—H22*B*⋯O61^v^	0.88	2.26	3.005 (4)	142
C32—H32⋯O11^vi^	0.95	2.47	3.399 (4)	165
N32—H32*A*⋯N61^vii^	0.88	2.14	2.965 (4)	156
N32—H32*B*⋯O11^vi^	0.88	2.14	2.952 (4)	153
C41—H41⋯O21^iv^	0.95	2.32	3.113 (4)	140
C42—H42⋯O61^iv^	0.95	2.63	3.547 (4)	163
N42—H42*A*⋯S1^iii^	0.88	2.68	3.523 (3)	161
N42—H42*B*⋯O61^iv^	0.88	2.22	3.063 (4)	159
N52—H52*A*⋯O41^viii^	0.88	2.09	2.921 (4)	157
N52—H52*B*⋯O61^ii^	0.88	2.06	2.882 (4)	155
C62—H62⋯S1^ix^	0.95	2.89	3.734 (3)	148
N62—H62*A*⋯O21	0.88	2.03	2.854 (4)	156
N62—H62*B*⋯O51^vi^	0.88	1.94	2.775 (4)	159
O71—H71⋯O31^ii^	0.84	2.20	3.020 (13)	167
O81—H81⋯O11^vi^	0.84	2.37	2.855 (7)	118
O81—H81⋯N32	0.84	2.58	3.062 (8)	118

Symmetry codes: (i) 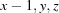; (ii) 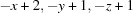; (iii) 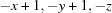; (iv) 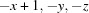; (v) 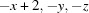; (vi) 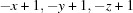; (vii) 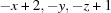; (viii) 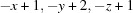; (ix) 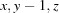.

**Table 2:** Experimental details

Crystal data	
Chemical formula	[Co(NCS)_2_(C_6_H_6_N_2_O)_4_]·2C_6_H_6_N_2_O·C_2_H_6_O
*M*_r_	953.92
Crystal system, space group	Triclinic, *P* 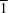
Temperature (K)	200
*a*, *b*, *c* (Å)	9.1877 (4), 13.6779 (5), 20.3185 (8)
α, β, γ (°)	104.027 (3), 97.256 (3), 109.576 (3)
*V* (Å^3^)	2273.12 (17)
*Z*	2
Radiation type	Mo *K*α
μ (mm^−1^)	0.53
Crystal size (mm)	0.42 × 0.35 × 0.25

Data collection	
Diffractometer	Stoe IPDS2
Absorption correction	Numerical (*X-SHAPE* and *X-RED32*; Stoe, 2008 ▸)
*T*_min_, *T*_max_	0.637, 0.805
No. of measured, independent and observed [*I* > 2σ(*I*)] reflections	23938, 9885, 8035
*R*_int_	0.044
(sin θ/λ)_max_ (Å^−1^)	0.639

Refinement	
*R*[*F* ^2^ > 2σ(*F* ^2^)], *wR*(*F* ^2^), *S*	0.054, 0.142, 1.10
No. of reflections	9885
No. of parameters	606
H-atom treatment	H-atom parameters constrained
Δρ_max_, Δρ_min_ (e Å^−3^)	0.75, −0.39

Computer programs: *X-AREA* (Stoe, 2008 ▸), *SHELXS97* (Sheldrick, 2008 ▸), *SHELXL2014* (Sheldrick, 2015 ▸), *XP* in *SHELXTL* (Sheldrick, 2008 ▸), *DIAMOND* (Brandenburg, 1999 ▸) and *publCIF* (Westrip, 2010 ▸).

## supplementary crystallographic information

### Crystal data

**Table:**

[Co(NCS)_2_(C_6_H_6_N_2_O)_4_]·2C_6_H_6_N_2_O·C_2_H_6_O	*Z* = 2
*M**_r_* = 953.92	*F*(000) = 990
Triclinic, *P*1	*D*_x_ = 1.394 Mg m^−^^3^
*a* = 9.1877 (4) Å	Mo *K*α radiation, λ = 0.71073 Å
*b* = 13.6779 (5) Å	Cell parameters from 9885 reflections
*c* = 20.3185 (8) Å	θ = 3.3–54.0°
α = 104.027 (3)°	µ = 0.53 mm^−^^1^
β = 97.256 (3)°	*T* = 200 K
γ = 109.576 (3)°	Block, red
*V* = 2273.12 (17) Å^3^	0.42 × 0.35 × 0.25 mm

### Data collection

**Table:**

Stoe IPDS-2 diffractometer	8035 reflections with *I* > 2σ(*I*)
ω scans	*R*_int_ = 0.044
Absorption correction: numerical (X-SHAPE and X-RED32; Stoe, 2008)	θ_max_ = 27.0°, θ_min_ = 1.7°
*T*_min_ = 0.637, *T*_max_ = 0.805	*h* = −11→10
23938 measured reflections	*k* = −17→17
9885 independent reflections	*l* = −24→25

### Refinement

**Table:**

Refinement on *F*^2^	0 restraints
Least-squares matrix: full	Hydrogen site location: inferred from neighbouring sites
*R*[*F*^2^ > 2σ(*F*^2^)] = 0.054	H-atom parameters constrained
*wR*(*F*^2^) = 0.142	*w* = 1/[σ^2^(*F*_o_^2^) + (0.0507*P*)^2^ + 3.0411*P*] where *P* = (*F*_o_^2^ + 2*F*_c_^2^)/3
*S* = 1.10	(Δ/σ)_max_ = 0.001
9885 reflections	Δρ_max_ = 0.75 e Å^−^^3^
606 parameters	Δρ_min_ = −0.39 e Å^−^^3^

### Special details

**Table:**

Geometry. All esds (except the esd in the dihedral angle between two l.s. planes) are estimated using the full covariance matrix. The cell esds are taken into account individually in the estimation of esds in distances, angles and torsion angles; correlations between esds in cell parameters are only used when they are defined by crystal symmetry. An approximate (isotropic) treatment of cell esds is used for estimating esds involving l.s. planes.

### Fractional atomic coordinates and isotropic or equivalent isotropic displacement parameters (Å^2^)

**Table:**

	*x*	*y*	*z*	*U*_iso_*/*U*_eq_	Occ. (<1)
Co1	0.61592 (5)	0.35492 (3)	0.21691 (2)	0.02592 (11)	
N1	0.8459 (3)	0.4542 (2)	0.22065 (14)	0.0336 (6)	
C1	0.9652 (4)	0.4826 (2)	0.20375 (15)	0.0304 (6)	
S1	1.13121 (10)	0.52353 (9)	0.17835 (5)	0.0467 (2)	
N2	0.3879 (3)	0.2543 (2)	0.21571 (14)	0.0361 (6)	
C2	0.2534 (4)	0.2022 (2)	0.20567 (15)	0.0307 (6)	
S2	0.06525 (11)	0.12699 (8)	0.18989 (5)	0.0497 (2)	
N11	0.5978 (3)	0.4880 (2)	0.29563 (13)	0.0297 (5)	
C11	0.4652 (4)	0.5085 (3)	0.29031 (16)	0.0357 (7)	
H11	0.3771	0.4615	0.2530	0.043*	
C12	0.4506 (4)	0.5955 (3)	0.33693 (17)	0.0363 (7)	
H12	0.3547	0.6080	0.3310	0.044*	
C13	0.5767 (4)	0.6641 (2)	0.39224 (16)	0.0302 (6)	
C14	0.7152 (4)	0.6439 (3)	0.39744 (16)	0.0335 (6)	
H14	0.8048	0.6895	0.4344	0.040*	
C15	0.7213 (4)	0.5563 (2)	0.34796 (16)	0.0316 (6)	
H15	0.8175	0.5442	0.3514	0.038*	
C16	0.5541 (4)	0.7534 (3)	0.44494 (16)	0.0335 (6)	
O11	0.4191 (3)	0.7524 (2)	0.44465 (12)	0.0401 (5)	
N12	0.6808 (4)	0.8311 (3)	0.49009 (18)	0.0560 (9)	
H12A	0.6701	0.8842	0.5211	0.067*	
H12B	0.7755	0.8297	0.4891	0.067*	
N21	0.6386 (3)	0.2271 (2)	0.13538 (13)	0.0307 (5)	
C21	0.5924 (4)	0.1226 (2)	0.13461 (16)	0.0312 (6)	
H21	0.5311	0.1002	0.1665	0.037*	
C22	0.6302 (4)	0.0461 (2)	0.08948 (16)	0.0319 (6)	
H22	0.5951	−0.0271	0.0906	0.038*	
C23	0.7195 (3)	0.0771 (2)	0.04279 (15)	0.0282 (6)	
C24	0.7621 (4)	0.1837 (2)	0.04143 (16)	0.0344 (7)	
H24	0.8198	0.2074	0.0088	0.041*	
C25	0.7195 (4)	0.2552 (2)	0.08825 (16)	0.0346 (7)	
H25	0.7494	0.3280	0.0868	0.041*	
C26	0.7649 (4)	−0.0066 (2)	−0.00419 (15)	0.0309 (6)	
O21	0.7255 (3)	−0.09870 (17)	0.00063 (12)	0.0410 (5)	
N22	0.8473 (4)	0.0237 (2)	−0.04995 (15)	0.0406 (6)	
H22A	0.8752	−0.0233	−0.0781	0.049*	
H22B	0.8739	0.0907	−0.0522	0.049*	
N31	0.7061 (3)	0.2929 (2)	0.29549 (13)	0.0303 (5)	
C31	0.6391 (4)	0.2855 (3)	0.34932 (16)	0.0313 (6)	
H31	0.5510	0.3067	0.3519	0.038*	
C32	0.6913 (4)	0.2485 (3)	0.40192 (16)	0.0318 (6)	
H32	0.6415	0.2460	0.4400	0.038*	
C33	0.8176 (4)	0.2152 (2)	0.39785 (15)	0.0300 (6)	
C34	0.8853 (4)	0.2197 (3)	0.34056 (17)	0.0365 (7)	
H34	0.9702	0.1957	0.3353	0.044*	
C35	0.8271 (4)	0.2594 (3)	0.29166 (16)	0.0348 (7)	
H35	0.8752	0.2634	0.2531	0.042*	
C36	0.8902 (4)	0.1787 (3)	0.45389 (16)	0.0335 (6)	
O31	1.0142 (3)	0.1628 (2)	0.44981 (13)	0.0444 (6)	
N32	0.8179 (4)	0.1652 (3)	0.50476 (16)	0.0490 (8)	
H32A	0.8575	0.1440	0.5379	0.059*	
H32B	0.7299	0.1774	0.5056	0.059*	
N41	0.5176 (3)	0.4126 (2)	0.13757 (13)	0.0306 (5)	
C41	0.3896 (4)	0.3416 (2)	0.08872 (16)	0.0353 (7)	
H41	0.3594	0.2664	0.0844	0.042*	
C42	0.2986 (4)	0.3719 (3)	0.04405 (16)	0.0346 (7)	
H42	0.2092	0.3183	0.0097	0.041*	
C43	0.3392 (3)	0.4808 (2)	0.05005 (15)	0.0279 (6)	
C44	0.4744 (4)	0.5551 (2)	0.09968 (17)	0.0325 (6)	
H44	0.5079	0.6307	0.1046	0.039*	
C45	0.5596 (4)	0.5178 (2)	0.14181 (16)	0.0319 (6)	
H45	0.6523	0.5694	0.1753	0.038*	
C46	0.2421 (4)	0.5236 (3)	0.00764 (15)	0.0313 (6)	
O41	0.2731 (3)	0.62182 (19)	0.02170 (13)	0.0428 (6)	
N42	0.1222 (3)	0.4499 (2)	−0.04437 (15)	0.0390 (6)	
H42A	0.0620	0.4713	−0.0701	0.047*	
H42B	0.1037	0.3801	−0.0529	0.047*	
N51	0.6258 (4)	0.9683 (2)	0.61213 (16)	0.0460 (7)	
C51	0.5455 (5)	1.0340 (3)	0.61289 (19)	0.0459 (8)	
H51	0.5017	1.0398	0.5698	0.055*	
C52	0.5234 (4)	1.0931 (3)	0.67293 (18)	0.0406 (7)	
H52	0.4627	1.1370	0.6710	0.049*	
C53	0.5905 (4)	1.0883 (3)	0.73626 (17)	0.0344 (6)	
C54	0.6730 (4)	1.0196 (3)	0.73642 (19)	0.0397 (7)	
H54	0.7194	1.0129	0.7788	0.048*	
C55	0.6854 (4)	0.9609 (3)	0.6724 (2)	0.0447 (8)	
H55	0.7398	0.9128	0.6722	0.054*	
C56	0.5693 (4)	1.1562 (3)	0.80151 (18)	0.0405 (7)	
O51	0.4479 (3)	1.1764 (3)	0.79954 (15)	0.0655 (9)	
N52	0.6846 (3)	1.1943 (2)	0.85867 (15)	0.0406 (6)	
H52A	0.6762	1.2358	0.8973	0.049*	
H52B	0.7693	1.1779	0.8578	0.049*	
N61	1.0108 (4)	−0.1655 (3)	0.36132 (16)	0.0445 (7)	
C61	1.0359 (4)	−0.2371 (3)	0.3113 (2)	0.0429 (8)	
H61	1.0812	−0.2845	0.3245	0.052*	
C62	1.0002 (4)	−0.2467 (3)	0.24140 (19)	0.0377 (7)	
H62	1.0214	−0.2988	0.2078	0.045*	
C63	0.9329 (3)	−0.1788 (2)	0.22133 (17)	0.0317 (6)	
C64	0.9026 (4)	−0.1056 (3)	0.27224 (17)	0.0355 (7)	
H64	0.8538	−0.0592	0.2602	0.043*	
C65	0.9444 (4)	−0.1010 (3)	0.34117 (19)	0.0429 (8)	
H65	0.9250	−0.0494	0.3759	0.051*	
C66	0.9038 (4)	−0.1824 (2)	0.14579 (15)	0.0292 (6)	
O61	0.9960 (3)	−0.20254 (18)	0.10943 (12)	0.0367 (5)	
N62	0.7810 (3)	−0.1622 (2)	0.12232 (14)	0.0365 (6)	
H62A	0.7600	−0.1624	0.0788	0.044*	
H62B	0.7194	−0.1484	0.1501	0.044*	
C71	0.678 (2)	0.5974 (12)	0.5795 (11)	0.106 (6)	0.4
H71A	0.5657	0.5518	0.5749	0.127*	0.4
H71B	0.7317	0.6140	0.6286	0.127*	0.4
C72	0.742 (3)	0.526 (3)	0.5390 (12)	0.095 (8)	0.4
H72A	0.7252	0.4627	0.5554	0.142*	0.4
H72B	0.6874	0.5028	0.4899	0.142*	0.4
H72C	0.8553	0.5657	0.5442	0.142*	0.4
O71	0.6780 (16)	0.6932 (13)	0.5727 (8)	0.122 (4)	0.4
H71	0.7702	0.7335	0.5731	0.182*	0.4
C81	0.9012 (14)	0.5036 (8)	0.5864 (8)	0.111 (4)	0.6
H81A	1.0100	0.5458	0.5836	0.133*	0.6
H81B	0.8927	0.5336	0.6346	0.133*	0.6
C82	0.802 (2)	0.531 (2)	0.5447 (13)	0.137 (10)	0.6
H82A	0.8318	0.6097	0.5598	0.205*	0.6
H82B	0.8111	0.5068	0.4963	0.205*	0.6
H82C	0.6926	0.4945	0.5478	0.205*	0.6
O81	0.8947 (8)	0.4053 (5)	0.5805 (5)	0.096 (2)	0.6
H81	0.8285	0.3619	0.5439	0.145*	0.6

### Atomic displacement parameters (Å^2^)

**Table:**

	*U*^11^	*U*^22^	*U*^33^	*U*^12^	*U*^13^	*U*^23^
Co1	0.0294 (2)	0.02770 (19)	0.02199 (19)	0.01389 (16)	0.00461 (15)	0.00611 (14)
N1	0.0332 (14)	0.0347 (13)	0.0328 (14)	0.0136 (11)	0.0090 (11)	0.0081 (11)
C1	0.0347 (16)	0.0326 (15)	0.0265 (14)	0.0175 (13)	0.0046 (12)	0.0083 (12)
S1	0.0337 (4)	0.0697 (6)	0.0498 (5)	0.0260 (4)	0.0163 (4)	0.0288 (5)
N2	0.0343 (14)	0.0376 (14)	0.0356 (14)	0.0141 (12)	0.0046 (11)	0.0108 (11)
C2	0.0342 (16)	0.0330 (15)	0.0262 (14)	0.0159 (13)	0.0082 (12)	0.0062 (12)
S2	0.0331 (4)	0.0516 (5)	0.0533 (5)	0.0102 (4)	0.0159 (4)	0.0018 (4)
N11	0.0324 (13)	0.0331 (13)	0.0239 (12)	0.0164 (11)	0.0043 (10)	0.0046 (10)
C11	0.0336 (16)	0.0433 (17)	0.0279 (15)	0.0198 (14)	0.0010 (12)	0.0025 (13)
C12	0.0338 (16)	0.0438 (17)	0.0324 (16)	0.0222 (14)	0.0035 (13)	0.0052 (13)
C13	0.0317 (15)	0.0335 (15)	0.0280 (14)	0.0158 (12)	0.0096 (12)	0.0076 (12)
C14	0.0315 (15)	0.0343 (15)	0.0309 (15)	0.0143 (13)	0.0042 (12)	0.0018 (12)
C15	0.0279 (14)	0.0366 (15)	0.0291 (15)	0.0160 (12)	0.0039 (12)	0.0040 (12)
C16	0.0351 (16)	0.0368 (16)	0.0313 (15)	0.0191 (13)	0.0081 (13)	0.0071 (13)
O11	0.0372 (12)	0.0475 (13)	0.0394 (13)	0.0247 (11)	0.0120 (10)	0.0059 (10)
N12	0.0380 (16)	0.0531 (18)	0.059 (2)	0.0232 (15)	0.0022 (14)	−0.0179 (15)
N21	0.0387 (14)	0.0302 (12)	0.0260 (12)	0.0180 (11)	0.0053 (10)	0.0077 (10)
C21	0.0348 (15)	0.0316 (15)	0.0303 (15)	0.0142 (12)	0.0100 (12)	0.0114 (12)
C22	0.0349 (15)	0.0271 (14)	0.0331 (15)	0.0111 (12)	0.0062 (13)	0.0098 (12)
C23	0.0326 (15)	0.0275 (13)	0.0221 (13)	0.0129 (12)	−0.0001 (11)	0.0043 (11)
C24	0.0494 (18)	0.0315 (15)	0.0247 (14)	0.0163 (14)	0.0112 (13)	0.0099 (12)
C25	0.0534 (19)	0.0260 (14)	0.0271 (15)	0.0166 (14)	0.0117 (14)	0.0095 (12)
C26	0.0344 (15)	0.0293 (14)	0.0258 (14)	0.0125 (12)	0.0018 (12)	0.0047 (11)
O21	0.0643 (16)	0.0288 (11)	0.0333 (12)	0.0224 (11)	0.0129 (11)	0.0078 (9)
N22	0.0535 (17)	0.0324 (13)	0.0403 (16)	0.0196 (13)	0.0216 (13)	0.0089 (12)
N31	0.0322 (13)	0.0379 (13)	0.0251 (12)	0.0176 (11)	0.0065 (10)	0.0110 (10)
C31	0.0301 (15)	0.0414 (16)	0.0276 (15)	0.0181 (13)	0.0084 (12)	0.0123 (12)
C32	0.0330 (15)	0.0416 (16)	0.0261 (14)	0.0173 (13)	0.0089 (12)	0.0140 (12)
C33	0.0306 (14)	0.0342 (15)	0.0263 (14)	0.0144 (12)	0.0052 (12)	0.0084 (12)
C34	0.0385 (17)	0.0498 (19)	0.0305 (16)	0.0278 (15)	0.0095 (13)	0.0117 (14)
C35	0.0370 (16)	0.0507 (18)	0.0258 (15)	0.0248 (15)	0.0125 (12)	0.0133 (13)
C36	0.0348 (16)	0.0392 (16)	0.0290 (15)	0.0186 (13)	0.0046 (12)	0.0096 (13)
O31	0.0434 (13)	0.0684 (16)	0.0370 (13)	0.0367 (13)	0.0098 (10)	0.0210 (12)
N32	0.0450 (17)	0.084 (2)	0.0408 (16)	0.0394 (17)	0.0153 (13)	0.0352 (17)
N41	0.0368 (13)	0.0313 (12)	0.0251 (12)	0.0172 (11)	0.0041 (10)	0.0064 (10)
C41	0.0502 (19)	0.0257 (14)	0.0261 (15)	0.0162 (13)	−0.0004 (13)	0.0030 (11)
C42	0.0422 (17)	0.0320 (15)	0.0250 (14)	0.0150 (13)	−0.0010 (13)	0.0040 (12)
C43	0.0324 (14)	0.0336 (14)	0.0232 (13)	0.0167 (12)	0.0107 (11)	0.0102 (11)
C44	0.0349 (16)	0.0276 (14)	0.0361 (16)	0.0114 (12)	0.0084 (13)	0.0122 (12)
C45	0.0311 (15)	0.0305 (14)	0.0310 (15)	0.0097 (12)	0.0042 (12)	0.0085 (12)
C46	0.0379 (16)	0.0378 (16)	0.0254 (14)	0.0198 (13)	0.0107 (12)	0.0126 (12)
O41	0.0587 (15)	0.0357 (12)	0.0384 (13)	0.0232 (11)	0.0045 (11)	0.0144 (10)
N42	0.0413 (15)	0.0409 (15)	0.0371 (15)	0.0212 (13)	−0.0002 (12)	0.0125 (12)
N51	0.0463 (17)	0.0402 (16)	0.0415 (17)	0.0134 (13)	0.0096 (14)	−0.0006 (13)
C51	0.052 (2)	0.048 (2)	0.0357 (18)	0.0201 (17)	0.0089 (16)	0.0080 (15)
C52	0.0428 (18)	0.0433 (18)	0.0354 (17)	0.0198 (15)	0.0057 (14)	0.0079 (14)
C53	0.0294 (15)	0.0380 (16)	0.0328 (16)	0.0112 (13)	0.0090 (12)	0.0070 (13)
C54	0.0361 (17)	0.0373 (17)	0.0421 (18)	0.0120 (14)	0.0060 (14)	0.0097 (14)
C55	0.0404 (18)	0.0334 (17)	0.054 (2)	0.0139 (15)	0.0079 (16)	0.0049 (15)
C56	0.0352 (17)	0.052 (2)	0.0369 (17)	0.0205 (15)	0.0128 (14)	0.0110 (15)
O51	0.0451 (15)	0.109 (3)	0.0438 (15)	0.0458 (17)	0.0086 (12)	0.0024 (16)
N52	0.0380 (15)	0.0519 (17)	0.0300 (14)	0.0213 (13)	0.0050 (12)	0.0045 (12)
N61	0.0411 (16)	0.0533 (17)	0.0388 (16)	0.0142 (14)	0.0039 (13)	0.0217 (14)
C61	0.0440 (19)	0.0446 (19)	0.046 (2)	0.0190 (16)	0.0059 (16)	0.0236 (16)
C62	0.0389 (17)	0.0339 (16)	0.0440 (18)	0.0154 (14)	0.0089 (14)	0.0166 (14)
C63	0.0259 (14)	0.0318 (15)	0.0349 (16)	0.0087 (12)	0.0021 (12)	0.0117 (12)
C64	0.0352 (16)	0.0432 (17)	0.0314 (16)	0.0195 (14)	0.0058 (13)	0.0118 (13)
C65	0.0422 (18)	0.051 (2)	0.0350 (17)	0.0186 (16)	0.0071 (14)	0.0112 (15)
C66	0.0314 (14)	0.0265 (13)	0.0289 (14)	0.0106 (12)	0.0056 (12)	0.0081 (11)
O61	0.0379 (12)	0.0409 (12)	0.0349 (12)	0.0194 (10)	0.0093 (10)	0.0109 (10)
N62	0.0386 (14)	0.0471 (15)	0.0296 (13)	0.0219 (13)	0.0083 (11)	0.0135 (12)
C71	0.125 (14)	0.056 (8)	0.129 (15)	0.016 (9)	0.034 (12)	0.038 (9)
C72	0.12 (2)	0.091 (15)	0.071 (12)	0.022 (16)	0.041 (14)	0.036 (11)
O71	0.108 (9)	0.155 (12)	0.136 (11)	0.059 (9)	0.054 (9)	0.077 (10)
C81	0.091 (7)	0.065 (6)	0.169 (13)	0.017 (5)	0.006 (8)	0.051 (7)
C82	0.096 (13)	0.088 (10)	0.167 (17)	−0.029 (9)	−0.051 (11)	0.070 (11)
O81	0.064 (4)	0.073 (4)	0.132 (7)	0.014 (3)	0.001 (4)	0.023 (4)

### Geometric parameters (Å, º)

**Table:**

Co1—N1	2.074 (3)	C41—H41	0.9500
Co1—N2	2.079 (3)	C42—C43	1.378 (4)
Co1—N31	2.179 (2)	C42—H42	0.9500
Co1—N11	2.181 (2)	C43—C44	1.391 (4)
Co1—N41	2.183 (2)	C43—C46	1.511 (4)
Co1—N21	2.185 (2)	C44—C45	1.384 (4)
N1—C1	1.160 (4)	C44—H44	0.9500
C1—S1	1.635 (3)	C45—H45	0.9500
N2—C2	1.162 (4)	C46—O41	1.227 (4)
C2—S2	1.631 (3)	C46—N42	1.338 (4)
N11—C11	1.337 (4)	N42—H42A	0.8800
N11—C15	1.339 (4)	N42—H42B	0.8800
C11—C12	1.387 (4)	N51—C55	1.319 (5)
C11—H11	0.9500	N51—C51	1.338 (5)
C12—C13	1.385 (4)	C51—C52	1.370 (5)
C12—H12	0.9500	C51—H51	0.9500
C13—C14	1.387 (4)	C52—C53	1.381 (5)
C13—C16	1.508 (4)	C52—H52	0.9500
C14—C15	1.388 (4)	C53—C54	1.391 (5)
C14—H14	0.9500	C53—C56	1.499 (4)
C15—H15	0.9500	C54—C55	1.393 (5)
C16—O11	1.236 (4)	C54—H54	0.9500
C16—N12	1.325 (4)	C55—H55	0.9500
N12—H12A	0.8800	C56—O51	1.235 (4)
N12—H12B	0.8800	C56—N52	1.330 (4)
N21—C25	1.336 (4)	N52—H52A	0.8800
N21—C21	1.343 (4)	N52—H52B	0.8800
C21—C22	1.384 (4)	N61—C61	1.333 (5)
C21—H21	0.9500	N61—C65	1.339 (5)
C22—C23	1.382 (4)	C61—C62	1.379 (5)
C22—H22	0.9500	C61—H61	0.9500
C23—C24	1.387 (4)	C62—C63	1.384 (4)
C23—C26	1.512 (4)	C62—H62	0.9500
C24—C25	1.383 (4)	C63—C64	1.381 (5)
C24—H24	0.9500	C63—C66	1.510 (4)
C25—H25	0.9500	C64—C65	1.385 (5)
C26—O21	1.221 (4)	C64—H64	0.9500
C26—N22	1.328 (4)	C65—H65	0.9500
N22—H22A	0.8800	C66—O61	1.247 (4)
N22—H22B	0.8800	C66—N62	1.309 (4)
N31—C31	1.330 (4)	N62—H62A	0.8800
N31—C35	1.341 (4)	N62—H62B	0.8800
C31—C32	1.388 (4)	C71—O71	1.351 (19)
C31—H31	0.9500	C71—C72	1.44 (4)
C32—C33	1.386 (4)	C71—H71A	0.9900
C32—H32	0.9500	C71—H71B	0.9900
C33—C34	1.392 (4)	C72—H72A	0.9800
C33—C36	1.515 (4)	C72—H72B	0.9800
C34—C35	1.375 (4)	C72—H72C	0.9800
C34—H34	0.9500	O71—H71	0.8400
C35—H35	0.9500	C81—O81	1.300 (11)
C36—O31	1.238 (4)	C81—C82	1.37 (2)
C36—N32	1.314 (4)	C81—H81A	0.9900
N32—H32A	0.8800	C81—H81B	0.9900
N32—H32B	0.8800	C82—H82A	0.9800
N41—C41	1.336 (4)	C82—H82B	0.9800
N41—C45	1.336 (4)	C82—H82C	0.9800
C41—C42	1.385 (4)	O81—H81	0.8400
			
N1—Co1—N2	178.32 (11)	C45—N41—Co1	123.6 (2)
N1—Co1—N31	90.01 (10)	N41—C41—C42	123.4 (3)
N2—Co1—N31	88.33 (10)	N41—C41—H41	118.3
N1—Co1—N11	89.42 (10)	C42—C41—H41	118.3
N2—Co1—N11	90.36 (10)	C43—C42—C41	119.2 (3)
N31—Co1—N11	92.30 (9)	C43—C42—H42	120.4
N1—Co1—N41	91.91 (10)	C41—C42—H42	120.4
N2—Co1—N41	89.74 (10)	C42—C43—C44	117.8 (3)
N31—Co1—N41	178.04 (10)	C42—C43—C46	123.6 (3)
N11—Co1—N41	88.09 (9)	C44—C43—C46	118.5 (3)
N1—Co1—N21	88.74 (10)	C45—C44—C43	119.3 (3)
N2—Co1—N21	91.53 (10)	C45—C44—H44	120.4
N31—Co1—N21	89.72 (9)	C43—C44—H44	120.4
N11—Co1—N21	177.27 (10)	N41—C45—C44	123.0 (3)
N41—Co1—N21	89.95 (9)	N41—C45—H45	118.5
C1—N1—Co1	158.0 (2)	C44—C45—H45	118.5
N1—C1—S1	178.5 (3)	O41—C46—N42	122.7 (3)
C2—N2—Co1	169.1 (3)	O41—C46—C43	120.3 (3)
N2—C2—S2	178.5 (3)	N42—C46—C43	117.0 (3)
C11—N11—C15	117.6 (3)	C46—N42—H42A	120.0
C11—N11—Co1	120.6 (2)	C46—N42—H42B	120.0
C15—N11—Co1	121.68 (19)	H42A—N42—H42B	120.0
N11—C11—C12	122.8 (3)	C55—N51—C51	117.6 (3)
N11—C11—H11	118.6	N51—C51—C52	123.1 (3)
C12—C11—H11	118.6	N51—C51—H51	118.4
C13—C12—C11	119.5 (3)	C52—C51—H51	118.4
C13—C12—H12	120.2	C51—C52—C53	119.2 (3)
C11—C12—H12	120.2	C51—C52—H52	120.4
C12—C13—C14	117.9 (3)	C53—C52—H52	120.4
C12—C13—C16	118.5 (3)	C52—C53—C54	118.4 (3)
C14—C13—C16	123.6 (3)	C52—C53—C56	118.5 (3)
C13—C14—C15	119.1 (3)	C54—C53—C56	123.1 (3)
C13—C14—H14	120.4	C53—C54—C55	117.8 (3)
C15—C14—H14	120.4	C53—C54—H54	121.1
N11—C15—C14	123.1 (3)	C55—C54—H54	121.1
N11—C15—H15	118.5	N51—C55—C54	123.7 (3)
C14—C15—H15	118.5	N51—C55—H55	118.1
O11—C16—N12	122.0 (3)	C54—C55—H55	118.1
O11—C16—C13	119.4 (3)	O51—C56—N52	122.9 (3)
N12—C16—C13	118.6 (3)	O51—C56—C53	118.8 (3)
C16—N12—H12A	120.0	N52—C56—C53	118.2 (3)
C16—N12—H12B	120.0	C56—N52—H52A	120.0
H12A—N12—H12B	120.0	C56—N52—H52B	120.0
C25—N21—C21	117.2 (3)	H52A—N52—H52B	120.0
C25—N21—Co1	118.8 (2)	C61—N61—C65	116.8 (3)
C21—N21—Co1	123.5 (2)	N61—C61—C62	124.0 (3)
N21—C21—C22	122.9 (3)	N61—C61—H61	118.0
N21—C21—H21	118.5	C62—C61—H61	118.0
C22—C21—H21	118.5	C61—C62—C63	118.6 (3)
C23—C22—C21	119.4 (3)	C61—C62—H62	120.7
C23—C22—H22	120.3	C63—C62—H62	120.7
C21—C22—H22	120.3	C64—C63—C62	118.3 (3)
C22—C23—C24	117.9 (3)	C64—C63—C66	122.2 (3)
C22—C23—C26	118.7 (3)	C62—C63—C66	119.4 (3)
C24—C23—C26	123.4 (3)	C63—C64—C65	119.0 (3)
C25—C24—C23	119.0 (3)	C63—C64—H64	120.5
C25—C24—H24	120.5	C65—C64—H64	120.5
C23—C24—H24	120.5	N61—C65—C64	123.3 (3)
N21—C25—C24	123.5 (3)	N61—C65—H65	118.4
N21—C25—H25	118.3	C64—C65—H65	118.4
C24—C25—H25	118.3	O61—C66—N62	123.4 (3)
O21—C26—N22	122.2 (3)	O61—C66—C63	119.9 (3)
O21—C26—C23	119.9 (3)	N62—C66—C63	116.7 (3)
N22—C26—C23	117.9 (3)	C66—N62—H62A	120.0
C26—N22—H22A	120.0	C66—N62—H62B	120.0
C26—N22—H22B	120.0	H62A—N62—H62B	120.0
H22A—N22—H22B	120.0	O71—C71—C72	127.2 (19)
C31—N31—C35	117.4 (3)	O71—C71—H71A	105.5
C31—N31—Co1	119.99 (19)	C72—C71—H71A	105.5
C35—N31—Co1	122.6 (2)	O71—C71—H71B	105.5
N31—C31—C32	123.3 (3)	C72—C71—H71B	105.5
N31—C31—H31	118.3	H71A—C71—H71B	106.1
C32—C31—H31	118.3	C71—C72—H72A	109.5
C33—C32—C31	118.8 (3)	C71—C72—H72B	109.5
C33—C32—H32	120.6	H72A—C72—H72B	109.5
C31—C32—H32	120.6	C71—C72—H72C	109.5
C32—C33—C34	118.2 (3)	H72A—C72—H72C	109.5
C32—C33—C36	123.5 (3)	H72B—C72—H72C	109.5
C34—C33—C36	118.3 (3)	C71—O71—H71	109.5
C35—C34—C33	118.9 (3)	O81—C81—C82	125.4 (14)
C35—C34—H34	120.6	O81—C81—H81A	106.0
C33—C34—H34	120.6	C82—C81—H81A	106.0
N31—C35—C34	123.4 (3)	O81—C81—H81B	106.0
N31—C35—H35	118.3	C82—C81—H81B	106.0
C34—C35—H35	118.3	H81A—C81—H81B	106.3
O31—C36—N32	123.0 (3)	C81—C82—H82A	109.5
O31—C36—C33	118.8 (3)	C81—C82—H82B	109.5
N32—C36—C33	118.2 (3)	H82A—C82—H82B	109.5
C36—N32—H32A	120.0	C81—C82—H82C	109.5
C36—N32—H32B	120.0	H82A—C82—H82C	109.5
H32A—N32—H32B	120.0	H82B—C82—H82C	109.5
C41—N41—C45	117.3 (3)	C81—O81—H81	109.5
C41—N41—Co1	118.0 (2)		

### Hydrogen-bond geometry (Å, º)

**Table:**

*D*—H···*A*	*D*—H	H···*A*	*D*···*A*	*D*—H···*A*
C11—H11···S1^i^	0.95	3.03	3.676 (3)	127
C14—H14···O31^ii^	0.95	2.62	3.532 (4)	162
C15—H15···O81^ii^	0.95	2.60	3.454 (7)	149
N12—H12*A*···N51	0.88	2.09	2.936 (4)	160
N12—H12*B*···O31^ii^	0.88	2.12	2.879 (4)	144
C25—H25···O41^iii^	0.95	2.47	3.100 (4)	124
N22—H22*A*···S2^iv^	0.88	2.60	3.439 (3)	160
N22—H22*B*···O61^v^	0.88	2.26	3.005 (4)	142
C32—H32···O11^vi^	0.95	2.47	3.399 (4)	165
N32—H32*A*···N61^vii^	0.88	2.14	2.965 (4)	156
N32—H32*B*···O11^vi^	0.88	2.14	2.952 (4)	153
C41—H41···O21^iv^	0.95	2.32	3.113 (4)	140
C42—H42···O61^iv^	0.95	2.63	3.547 (4)	163
N42—H42*A*···S1^iii^	0.88	2.68	3.523 (3)	161
N42—H42*B*···O61^iv^	0.88	2.22	3.063 (4)	159
N52—H52*A*···O41^viii^	0.88	2.09	2.921 (4)	157
N52—H52*B*···O61^ii^	0.88	2.06	2.882 (4)	155
C62—H62···S1^ix^	0.95	2.89	3.734 (3)	148
N62—H62*A*···O21	0.88	2.03	2.854 (4)	156
N62—H62*B*···O51^vi^	0.88	1.94	2.775 (4)	159
O71—H71···O31^ii^	0.84	2.20	3.020 (13)	167
O81—H81···O11^vi^	0.84	2.37	2.855 (7)	118
O81—H81···N32	0.84	2.58	3.062 (8)	118

Symmetry codes: (i) *x*−1, *y*, *z*; (ii) −*x*+2, −*y*+1, −*z*+1; (iii) −*x*+1, −*y*+1, −*z*; (iv) −*x*+1, −*y*, −*z*; (v) −*x*+2, −*y*, −*z*; (vi) −*x*+1, −*y*+1, −*z*+1; (vii) −*x*+2, −*y*, −*z*+1; (viii) −*x*+1, −*y*+2, −*z*+1; (ix) *x*, *y*−1, *z*.
